# A systematic comparison of ATN biomarkers for monitoring longitudinal cognitive changes in Alzheimer's disease

**DOI:** 10.1002/alz.70783

**Published:** 2025-10-22

**Authors:** Davina Biel, Anna Steward, Anna Dewenter, Amir Dehsarvi, Zeyu Zhu, Sebastian N Roemer‐Cassiano, Lukas Frontzkowski, Fabian Hirsch, Carla Palleis, Günter Höglinger, Matthias Brendel, Nicolai Franzmeier, for the Alzheimer's Disease Neuroimaging Initiative (ADNI)

**Affiliations:** ^1^ Institute for Stroke and Dementia Research (ISD) University Hospital, LMU Munich Munich Germany; ^2^ Department of Neurology University Hospital, LMU Munich Munich Germany; ^3^ Department of Nuclear Medicine University Hospital, LMU Munich Munich Germany; ^4^ German Center for Neurodegenerative Diseases (DZNE) Munich Germany; ^5^ Munich Cluster for Systems Neurology (SyNergy) Munich Germany; ^6^ The Sahlgrenska Academy, Institute of Neuroscience and Physiology Department of Psychiatry and Neurochemistry, Mölndal and Gothenburg University of Gothenburg Mölndal and Gothenburg Sweden

**Keywords:** Alzheimer's disease, amyloid‐PET, cortical thickness, p‐tau, plasma phosphorylated tau, tau‐PET, treatment monitoring

## Abstract

**INTRODUCTION:**

With anti‐amyloid beta (Aβ) therapies approved for Alzheimer's disease (AD), surrogate biomarkers are needed to monitor clinical treatment efficacy. Therefore, we systematically compared longitudinal changes in A/T/N biomarkers (amyloid‐positron emission tomography [PET], tau‐PET, plasma phosphorylated tau at threonine 217 [p‐tau_217_], and magnetic resonance imaging) for tracking cognitive changes.

**METHODS:**

We analyzed longitudinal biomarker and cognitive change rates from the Alzheimer's Disease Neuroimaging Initiative (*N *= 141) and Anti‐Amyloid Treatment in Asymptomatic Alzheimer's (A4) and Longitudinal Evaluation of Amyloid Risk and Neurodegeneration (LEARN) (*N* = 151), estimated using linear mixed models. Using linear models, we tested biomarker changes as predictors of cognitive changes, comparing predictive strengths across biomarkers using bootstrapping.

**RESULTS:**

Tau‐PET, plasma p‐tau217, and cortical thickness changes accurately tracked change rates in Mini‐Mental State Examination, Alzheimer's Disease Assessment Scale‐Cognitive Subscale 13‐item version, Clinical Dementia Rating‐Sum of Boxes, and Preclinial Alzheimer Cognitive Composite scores. In contrast, amyloid‐PET change rates were not linked to cognitive changes.

**DISCUSSION:**

Plasma p‐tau_217_ offers a cost‐effective AD‐specific alternative to tau‐PET and could potentially be implemented for monitoring cognitive changes in AD trials, while amyloid‐PET lacks utility. Cortical thickness changes accurately track cognitive changes but may be confounded by pseudo‐atrophy in anti‐Aβ treatments.

**Highlights:**

- Longitudinal changes in tau‐PET, plasma p‐tau_217_, cortical thickness – but not amyloid‐PET – effectively track cognitive decline.
- Cortical thickness may be confounded by pseudo‐atrophy in anti‐Aβ trials.
- Plasma p‐tau_217_ is a robust and cost‐effective alternative to tau‐PET as an AD‐specific surrogate biomarker for monitoring cognitive changes.

## 1 BACKGROUND

Alzheimer's disease (AD) is characterized by amyloid beta (Aβ) plaques that trigger the aggregation of neurofibrillary tau tangles, leading to neuronal loss and cognitive decline.^1^ Importantly, Aβ plaques that drive the amyloid cascade of AD emerge over many years before symptom manifestation,^2^, ^3^ posing significant challenges for timely diagnosis and treatment. With the recent US Food and Drug Administration (FDA) and EMA approval of the first disease‐modifying anti‐Aβ drugs that clear cerebral Aβ plaque pathology,^4^, ^5^, ^6^ reliable biomarkers to track disease progression and treatment efficacy are urgently needed. While assessing the natural progression of AD via Aβ, tau, and neurodegeneration (i.e., A/T/N) biomarkers is largely established,^7^ identifying the most suitable biomarker for tracking longitudinal cognitive changes – and thereby potentially assessing clinical treatment efficacy – remains unresolved.

Amyloid‐positron emission tomography (PET), which measures fibrillar Aβ deposition, a defining feature of AD, has long been a cornerstone in AD research and clinical trials. It plays a critical role in confirming fibrillar Aβ presence,^8^ which is crucial for participant selection in anti‐Aβ trials. Many clinical trials thus use Aβ reduction as a target engagement measure in conjunction with cognitive assessments to evaluate treatment efficacy.^9^, ^10^ However, Aβ accumulation begins decades before symptom onset and plateaus in later disease stages,^2^ making it suboptimal for tracking short‐term cognitive changes^11^ or clinical treatment efficacy beyond target engagement (i.e., Aβ clearance). As anti‐Aβ therapies aim to slow or halt clinical progression, biomarkers that better reflect cognitive decline are needed as surrogate biomarkers of clinical treatment efficacy in real‐world settings. Tau‐PET imaging, which measures neurofibrillary tangle accumulation, strongly associates with cognitive decline.^11^, ^12^ Unlike Aβ, tau pathology spreads in a disease‐stage‐dependent manner,^13^ closely paralleling symptom severity.^11^, ^14^, ^15^ This makes tau‐PET a more promising biomarker for tracking cognitive status in AD than amyloid‐PET. Similarly, plasma phosphorylated tau at threonine 217 (p‐tau_217_) reflects tau pathophysiology with high specificity for AD^16^, ^17^ and tracks disease progression by increasing dynamically as AD advances.^18^ Elevated plasma p‐tau_217_ levels correlate strongly with amyloid‐PET,^18^, ^19^, ^20^ tau‐PET,^19^, ^20^ and cerebrospinal fluid (CSF) p‐tau_217_,^21^ indicating its suitability as a peripheral and easy‐to‐obtain AD biomarker. Compared to imaging or CSF‐based biomarkers, plasma p‐tau_217_ offers practical advantages, including lower cost, increased accessibility, and ease of repeated sampling, enabling more frequent and less invasive treatment monitoring in clinical trials and real‐world settings. In addition to PET and fluid biomarkers, structural magnetic resonance imaging (MRI)‐based measures, such as cortical thickness, have been widely used to track AD‐related neurodegeneration.^22^ Particularly in AD‐vulnerable regions, cortical thickness correlates with cognition^23^, ^24^ and serves as a neuroimaging biomarker to predict future cognitive decline.^25^, ^26^

**RESEARCH IN CONTEXT**

1. **Systematic review**: With the approval of anti‐Aβ drugs for AD, surrogate biomarkers are needed to track cognitive changes for treatment monitoring. While cross‐sectional analyses of A/T/N (amyloid/tau/neurodegeneration) biomarkers and cognitive decline are common, longitudinal analysis of A/T/N trajectories may better capture the clinical progression of AD.
2. **Interpretation**: A systematic comparison of A/T/N biomarkers showed that changes in tau‐PET, plasma p‐tau_217_, and MRI‐assessed cortical thickness consistently tracked cognitive changes across two AD cohorts, whereas amyloid‐PET changes did not. The high costs of tau‐PET and limitations of cortical thickness in the context of anti‐Aβ treatment position plasma p‐tau_217_ as a cost‐effective, accessible alternative to track clinical AD progression.
3. **Future directions**: Although Aβ is critical for assessing target engagement in anti‐Aβ trials, its weak link to cognition limits its utility for tracking clinical benefits. Plasma p‐tau_217_ may be integrated into clinical trial designs for AD to enhance treatment monitoring beyond Aβ clearance.

While cross‐sectional biomarker assessments are essential for AD diagnosis, staging, and prognosis of future cognitive decline,^11^, ^27^ they reflect only a single‐time‐point snapshot of disease status.^11^, ^27^, ^28^ In contrast, longitudinal biomarker changes provide a more dynamic view of disease progression and may better capture treatment‐related cognitive effects.^2^ However, although cross‐sectional analyses of biomarkers have been widely used to study associations between biomarkers and cognitive decline,^11^, ^29^ longitudinal changes across A/T/N biomarkers have not yet been systematically compared for their ability to track cognitive decline.

Therefore, the main aim of the current study was to address this gap by directly comparing longitudinal A/T/N biomarker trajectories (i.e., amyloid‐PET, tau‐PET, plasma p‐tau_217_, and MRI‐derived cortical thickness) to determine which biomarker best tracked cognitive decline in AD. These insights will help evaluate their potential as surrogate biomarkers for assessing treatment efficacy in anti‐Aβ clinical settings. To this end, we included data from two well‐characterized cohorts – the Alzheimer's Disease Neuroimaging Initiative (ADNI) and the Anti‐Amyloid Treatment in Asymptomatic Alzheimer's (A4) and Longitudinal Evaluation of Amyloid Risk and Neurodegeneration (LEARN) study with comprehensive longitudinal biomarker and clinical characterization. The results of this study will help optimize the dynamic use of biomarkers to capture clinical decline, thereby supporting their use as surrogate endpoints for evaluating treatment efficacy in AD.

## 2 METHODS

### 2.1 Participants

To assess whether AD biomarkers tracked cognitive changes, we leveraged 141 participants from the ADNI cohort with available longitudinal (> 1 measurement) data of ^18^F‐florbetapir/^18^F‐florbetaben amyloid‐PET, ^18^F‐flortaucipir tau‐PET, plasma p‐tau_217_, brain atrophy measures by MRI together with longitudinal cognitive assessments (Mini‐Mental State Examination [MMSE], Alzheimer's Disease Assessment Scale‐Cognitive Subscale 13‐item version [ADAS13], and Clinical Dementia Rating [CDR]‐Sum of Boxes [SB]), demographic information (sex, age, education, and *ApoE4*), and clinical status. Baseline data had to be collected within 6 months.^30^, ^31^ Clinical status was definined as cognitively normal (CN; MMSE ≥ 24, CDR = 0, non‐depressed), mild cognitive impairment (mild cognitive impairment [MCI]; MMSE ≥ 24, CDR = 0.5, objective memory impairment on education‐adjusted Wechsler Memory Scale II, preserved Activities of Daily Living), or dementia (MMSE = 20 to 26, CDR ≥ 0.5, National Institute of Neurological and Communicative Disorders/Alzheimer's Disease and Related Disorders Association criteria for probable AD).

In addition, we included 151 participants from the A4/LEARN studies. As for ADNI, eligible participants had available longitudinal (> 1 measurement) data of ^18^F‐florbetapir amyloid‐PET, ^18^F‐flortaucipir tau‐PET, plasma p‐tau_217_, and brain atrophy measures by MRI together with longitudinal cognitive assessments (MMSE, Preclinical Alzheimer Cognitive Composite [PACC]), and demographic information (sex, age, education). The A4/LEARN studies included cognitively normal participants (CN; MMSE ≥ 25, CDR = 0, Logical Memory II score of 6 to 18 at baseline). For more details see A4 (https://clinicaltrials.gov/study/NCT02008357) and LEARN (https://www.clinicaltrials.gov/study/NCT02488720) inclusion criteria. In both cohorts, Aβ status (−/+) was determined using tracer‐specific cut‐offs for global amyloid‐PET (i.e., Aβ+ = standardized uptake value ratio [SUVR] > 1.11/1.08 for ^8^F‐florbetapir^32^/^18^F‐florbetaben^33^). All study procedures were in accordance with the Declaration of Helsinki. Ethical approval was obtained by ADNI and A4/LEARN investigators, and all study participants provided written informed consent.

### 2.2 Cognitive assessments

We included the MMSE,^34^ ADAS13,^35^ and CDR‐SB^36^ for ADNI and the MMSE and PACC^37^ for the A4/LEARN cohort. These widely used cognitive and functional assessments in AD clinical trials evaluate key domains such as memory, language, executive function, and functional abilities, with the PACC being more sensitive in the early preclinical stages of AD, that is, the target group included in the A4/LEARN sample.^38^ Importantly, the MMSE,^39^ ADAS13,^39^ CDR‐SB,^40^ and PACC^37^ are commonly used as primary or secondary endpoints in AD clinical trials to track cognitive changes, assess treatment efficacy, and are well validated and standardized across AD research, ensuring comparability across studies and regulatory agencies (e.g., FDA and EMA).

### 2.3 Plasma p‐tau_217_ assessment

The ADNI biomarker core team at the University of Pennsylvania collected blood samples in EDTA collection tubes used to obtain plasma following the ADNI4 Procedures manual version 2.0 (https://adni.loni.usc.edu/wp‐content/uploads/2024/02/ADNI4_Procedures_Manual_v2.0.pdf). After the samples were stored at −80°C until the day of the analysis, plasma p‐tau_217_ samples were analyzed using immunoassay reagents provided by Fujirebio on the validated and automated Lumipulse G1200 chemiluminescent enzyme immunoassay platform. The data are provided in the ADNI database in the “UPENN_PLASMA_FUJIREBIO_QUANTERIX” file.

For the A4/LEARN cohort, plasma p‐tau_217_ was analyzed on a validated electrochemiluminescence immunoassay by Eli Lilly and Company using a MesoScale Discovery (MSD) Sector S Imager 600 MM at the Lilly Clinical Diagnostics Laboratory.^41^

### 2.4 MRI and PET acquisition and preprocessing

ADNI acquired 3T structural magnetic resonance images of T1‐weighted scans using MPRAGE sequences (https://adni.loni.usc.edu/wp‐content/uploads/2017/07/ADNI3‐MRI‐protocols.pdf). Amyloid‐PET was recorded 50 to 70 min after ^18^F‐florbetapir injection in 4 × 5 min frames or 90 to 110 min after ^18^F‐florbetaben injection in 4 × 5 min frames. Tau‐PET was recorded 75 to 105 min after ^18^F‐flortaucipir injection in 6 × 5 min frames. To obtain mean images, the recorded time frames were motion corrected and averaged (for more information see: https://adni.loni.usc.edu/help‐faqs/adni‐documentation/). The imaging protocols of A4/LEARN were congruent with those of ADNI.

After images were screened for artifacts, preprocessing was performed independently for ADNI and A4/LEARN using a uniform processing pipeline. Using the CAT12 toolbox (https://neuro‐jena.github.io/cat12‐help/), T1‐weighted scans were bias‐corrected, segmented, and non‐linearly warped to Montreal Neurological Institute (MNI) space. Cortical thickness was assessed using the longitudinal CAT12 cortical thickness pipeline, from which a pre‐established AD summary region based on the Desikan–Killiany atlas was used to track AD‐related neurodegeneration patterns (i.e., including entorhinal, fusiform, inferior temporal, and middle temporal thickness measures).^42^ PET images acquired dynamically were realigned and averaged into single images, which were then rigidly aligned to the T1‐weighted MRI scan. Amyloid‐PET SUVRs were intensity normalized to the whole cerebellum^43^ and tau‐PET SUVRs to the inferior cerebellar gray matter.^44^ Regional SUVRs for tau‐PET were determined for the 200 regions of the cortical Schaefer atlas.^45^ To harmonize between amyloid‐PET tracers, global SUVRs of ^18^F‐florbetapir and ^18^F‐florbetaben were transformed to the Centiloid scale using equations provided by ADNI.^46^ A temporal meta region of interest (ROI) for tau‐PET SUVRs, which was previously shown to capture AD‐related tau accumulation and cognitive decline,^28^, ^47^ was created using Desikan–Killiany atlas‐based SUVR data following the ADNI guidelines.

### 2.5 Statistical analyses

To assess whether changes in AD biomarkers (i.e., Centiloid, temporal tau‐PET, plasma p‐tau_217_, or cortical thickness of the AD signature region) tracked changes in cognition, we first calculated change rates for each A/T/N biomarker and cognitive scores. To that end, we used linear mixed models with time (i.e., years from baseline) as independent variable and Centiloid, tau‐PET SUVRs, plasma p‐tau_217_, or cortical thickness as the respective dependent variable, incorporating random intercepts and slopes to account for individual variability.^30^ For A4/LEARN, Centiloid change rates were estimated using a linear model rather than a linear mixed model, as only one follow‐up measurement was available per patient. To generate cognitive endpoints, we determined cognitive changes over time. As above, we fitted linear mixed models with time as independent variable and scores of the MMSE, ADAS13, CDR‐SB, or PACC as the dependent variable, with random intercepts and slopes.^48^ Overall, this statistical approach allows standardizing change rates to a commonly interpretable metric (i.e., change per year) regardless of different overall follow‐up times. In a second step, we calculated linear regression models, using the before calculated change rates of AD biomarkers as independent variables, and change rates in cognition as dependent variables, controlling for age, sex, education, baseline cognitive scores, and maximum follow‐up times per subject, with ADNI models additionally adjusted for clinical status given the inclusion of CN, MCI, and dementia subjects in this cohort. Importantly, the computation of cognitive change rates was matched to each biomarker modality by using only overlapping cognitive and biomarker follow‐up data.

To further compare the strengths of correlations between biomarkers and cognitive decline, we performed bootstrapped linear regression with 1000 iterations for each cognitive test within each cohort. Within each iteration, standardized beta estimates for the association between biomarkers (i.e., Centiloid, tau‐PET, plasma p‐tau_217_, and cortical thickness) and cognitive changes of the respective test were extracted. As for the linear models, biomarker changes were included as independent and cognitive changes as dependent variables, controlling for age, sex, education, and baseline cognitive scores, with ADNI models additionally adjusted for clinical status. For non‐parametric comparisons, we used the percentile method to calculate 95% confidence intervals (CIs) for standardized beta values. If a 95% CI crossed zero, it indicated that the predictor might not have a statistically significant association with the outcome. To assess standardized differences of the predictive strength between biomarker modalities, effect sizes were calculated using Cohen's *d* using absolute standardized beta values of the association between biomarker modality and cognitive changes. All statistical analyses were performed using R statistical software version 4.3.1 (http://www.R‐project.org).^49^

### 2.6 Data availability

All data used in this manuscript are publicly available from the ADNI (adni.loni.usc.edu) or A4/LEARN (a4studydata.org) database upon registration and compliance with the data use agreement. The processed datasets that support the findings of this study are available upon reasonable request from the corresponding author and upon proving registration and compliance agreements with the ADNI and A4/LEARN databases.

## 3 RESULTS

### 3.1 Sample characteristics

**FIGURE 1:**
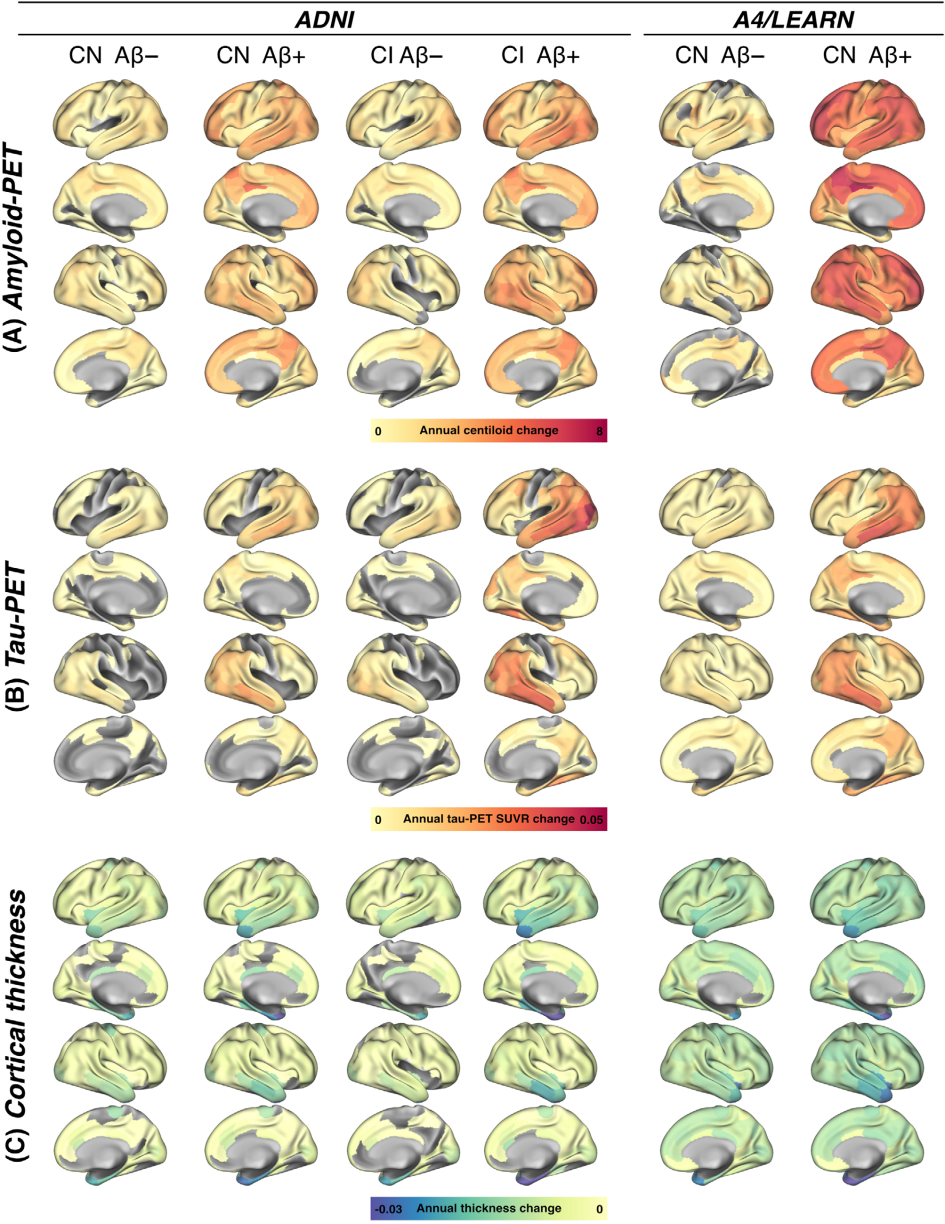
Surface rendering of regional change rates in (A) amyloid‐PET, (B) tau‐PET, and (C) MRI‐assessed cortical thickness, stratified by clinical (i.e., cognitively normal = CN, cognitively impaired = CI) and amyloid‐PET status.

To evaluate whether longitudinal changes in AD biomarkers (Centiloid, tau‐PET, plasma p‐tau_217_, and cortical thickness) tracked cognitive decline, we included 141 participants (i.e., 94/38/9 CN/MCI/dementia) from the ADNI cohort who also had available longitudinal cognitive assessments for MMSE, ADAS13, and CDR‐SB. Similarly, we included 151 subjects from the A4/LEARN cohort with longitudinal biomarker (i.e., Centiloid, tau‐PET, plasma p‐tau_217_, and cortical thickness) and cognitive assessments (i.e., MMSE and PACC). Mean follow‐up times per biomarker and biomarker‐matched cognitive follow‐up data are shown in Table 1. Surface renderings of annual change rates in imaging biomarker data (i.e., amyloid‐PET, tau‐PET, and MRI) are shown in Figure 1.

**TABLE 1:** Sample characteristics.

ADNI (*n *= 141)	ADNI (*n* = 141)	A4/LEARN (*n* = 151)
Clinical status (CN/MCI/Dem)	94/38/9	151/0/0
Amyloid‐PET status (Aβ−/Aβ+)	67/74	30/121
Sex (male/female)	69/72	59/92
Age	76.81 ± 7.04	70.61 ± 4.36
Centiloid	33.13 ± 37 to 25	54.34 ± 34.47
Centiloid ROC	1.82 ± 2.58	4.19 ± 5.06
Centiloid + Cognition follow‐up years	6.13 ± 2.21	4.62 ± 0
Centiloid‐matched MMSE ROC	−0.13 ± 0.28	−0.09 ± 0.48
Centiloid‐matched ADAS13 ROC	0.61 ± 0.72	
Centiloid‐matched CDR‐SB ROC	0.12 ± 0.28	
Centiloid‐matched PACC ROC		−0.25 ± 0.99
Temporal meta tau‐PET SUVR	1.21 ± 0.18	1.17 ± 0.13
Temporal meta tau‐PET SUVR ROC	0.01 ± 0.02	0.02 ± 0.02
Tau‐PET + Cognition follow‐up years	2.78 ± 1.56	4.83 ± 0.98
Tau‐PET‐matched MMSE ROC	−0.2 ± 0.21	−0.08 ± 0.25
Tau‐PET‐matched ADAS13 ROC	0.69 ± 0.67	
Tau‐PET‐matched CDR‐SB ROC	0.18 ± 0.35	
Tau‐PET‐matched PACC ROC		−0.21 ± 0.79
MRI cortical thickness AD meta ROI	2.83 ± 0.14	2.87 ± 0.12
MRI cortical thickness AD meta ROI ROC	−0.01 ± 0.01	−0.02 ± 0.02
MRI + Cognition follow‐up years	5.71 ± 2.49	5.18 ± 0.56
MRI‐matched MMSE ROC	−0.1 ± 0.32	−0.12 ± 0.35
MRI‐matched ADAS13 ROC	0.61 ± 0.78	
MRI‐matched CDR‐SB ROC	0.12 ± 0.28	
MRI‐matched PACC ROC		−0.31 ± 0.88
P‐tau_217_	0.17 ± 0.18	0.24 ± 0.13
P‐tau_217_ ROC	0.02 ± 0.02	0.02 ± 0.04
P‐tau_217_ + Cognition follow‐up years	4.35 ± 2.03	4.13 ± 1.32
P‐tau_217_‐matched MMSE ROC	−0.13 ± 0.26	−0.11 ± 0.3
P‐tau_217_‐matched ADAS13 ROC	0.55 ± 0.57	
P‐tau_217_‐matched CDR‐SB ROC	0.12 ± 0.33	
P‐tau_217_‐matched PACC ROC		−0.28 ± 0.87

Abbreviations: Aβ, amyloid beta; AD, Alzheimer's disease; ADAS13, Alzheimer's Disease Assessment Scale‐Cognitive Subscale 13‐item version; ADNI, Alzheimer's Disease Neuroimaging Initiative; CDR‐SB, Clinical Dementia Rating ‐ sum of boxes; CN, cognitively normal; Dem, dementia; MCI, mild cognitive impairment; MMSE, Mini‐Mental State Examination; MRI, magnetic resonance imaging; PACC, Preclinical Alzheimer's Cognitive Composite; PET, positron emission tomography; p‐tau_217_, phosphorylated tau at threonine 217; ROC, rate of change;SUVR, standardized uptake value ratio.

### 3.2 Changes in tau‐PET, plasma p‐tau_217_, and cortical thickness but not amyloid‐PET track cognitive decline

**FIGURE 2:**
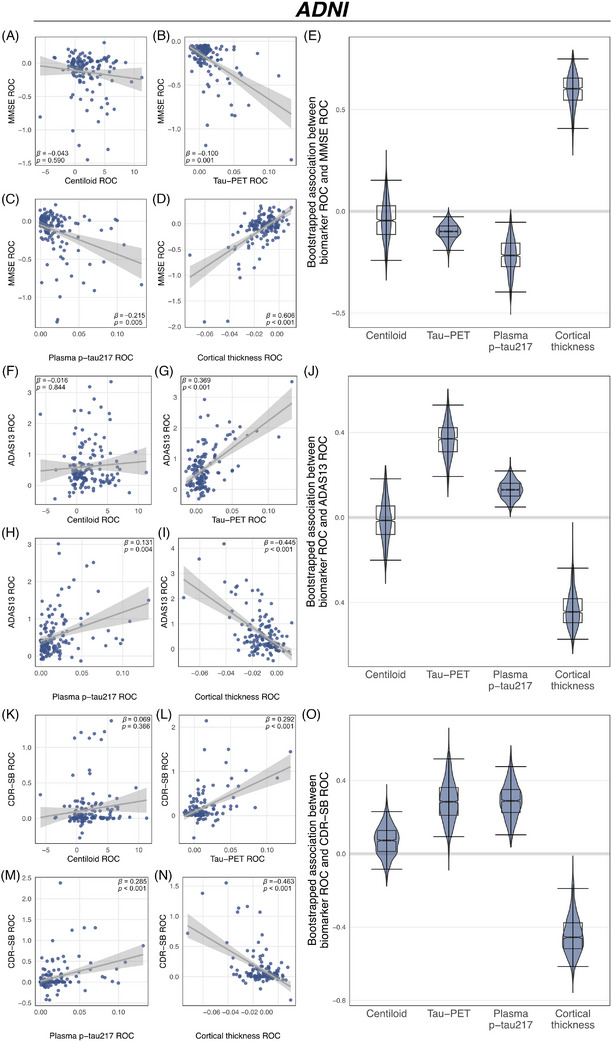
Comparing the link between biomarker dynamics and cognitive decline within the ADNI cohort. Rates of changes (ROC) of amyloid‐PET (in centiloid), tau‐PET, plasma p‐tau_217_ and cortical thickness were used to track cognitive changes in the MMSE (A)–(D), ADAS13 (F)–(I), and CDR‐SB (K)–(N) using linear regression. Plots display standardized beta values (β) and p‐values. Bootstrapped models with 95% confidence intervals were used to compare standardized beta values of the association between biomarker changes and changes in the MMSE (E), ADAS13 (J), and the CDR‐SB (O). The models are controlled for sex, age, education, clinical status, and baseline cognition.

**FIGURE 3:**
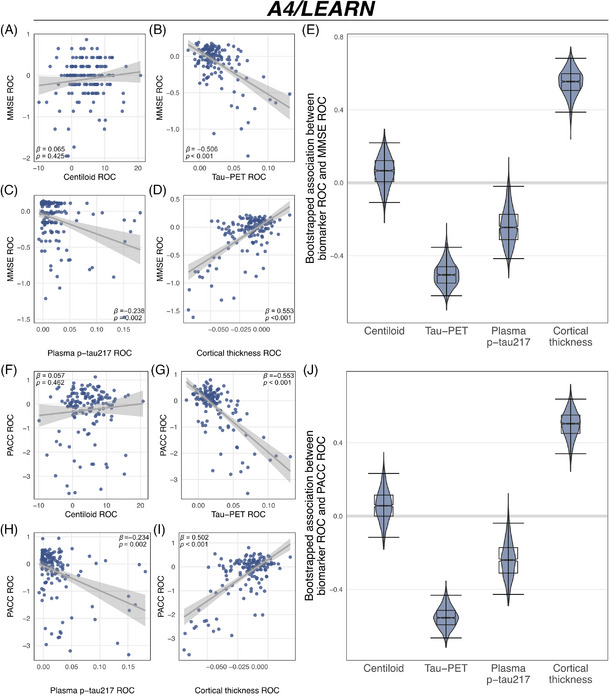
Comparing the link between biomarker dynamics and cognitive decline within the A4/LEARN cohort. Rates of changes (ROC) of amyloid‐PET (in centiloid), tau‐PET, plasma p‐tau_217_ and cortical thickness were used to track cognitive changes in the MMSE (A)–(D) and PACC (F)–(I) using linear regression. Plots display standardized beta values (β) and p‐values. Bootstrapped models with 95% confidence intervals were used to compare standardized beta values of the association between biomarker changes and changes in the MMSE (E) and PACC (J). The models are controlled for sex, age, education, and baseline cognition.

**FIGURE 4:**
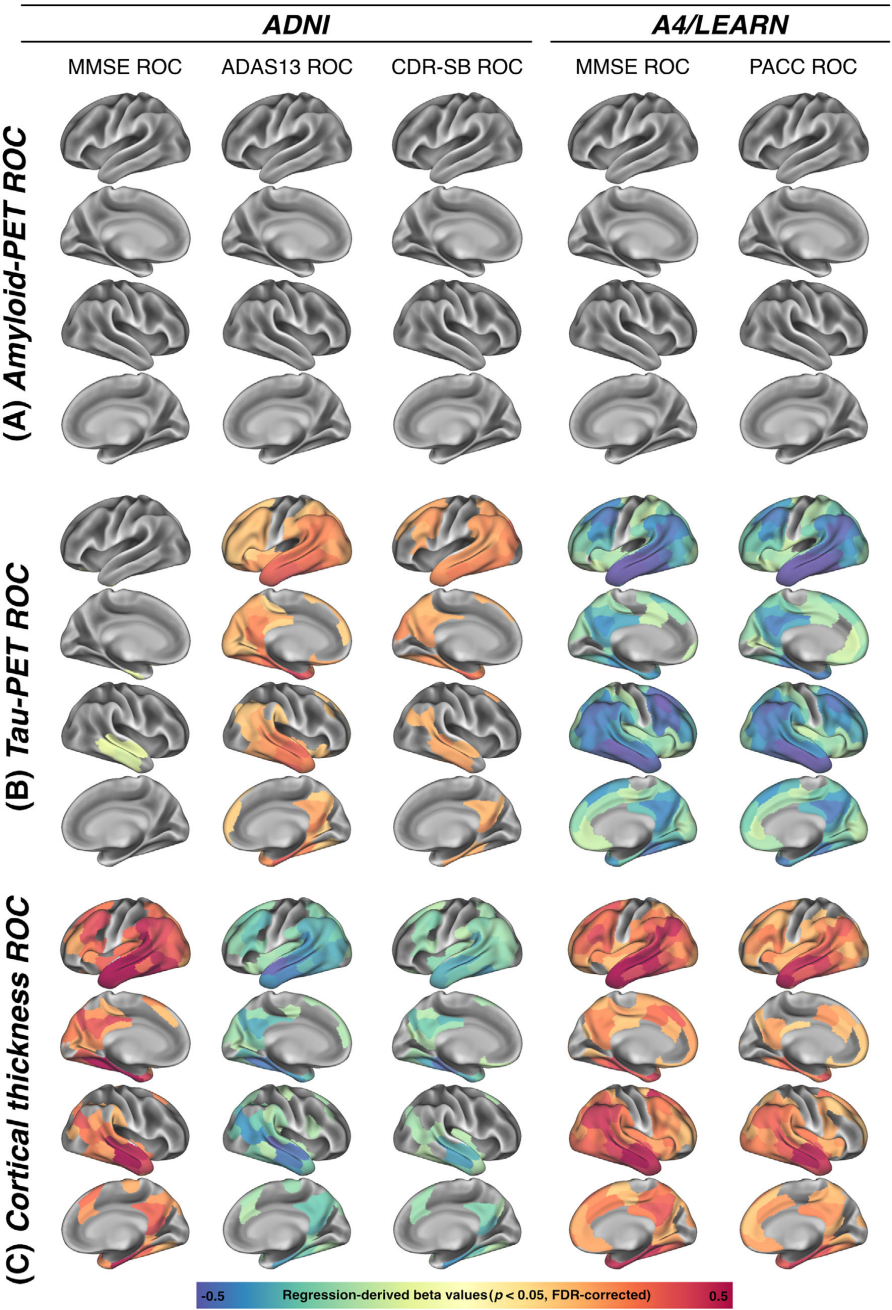
ROI‐based regression models, illustrating the associations between annual change rates of (A) amyloid‐PET centiloids, (B) tau‐PET or (C) MRI‐assessed cortical thickness with each cognitive test in ADNI and A4/LEARN. Standardized regression coefficients that were significant (*p* < 0.05) after False‐discovery rate (FDR) correction are displayed. All models are controlled for sex, age, education, clinical status (in ADNI), maximum follow up‐duration, baseline cognition and total intracranial volume for cortical thickness analyses.

We first examined whether changes in Centiloid, temporal meta‐ROI tau‐PET, plasma p‐tau_217_, and MRI‐assessed cortical thickness in the AD meta‐ROI (entorhinal, fusiform, inferior temporal, and middle temporal cortex)^42^ were associated with cognitive decline. In ADNI, changes in Centiloid were not associated with changes in MMSE (*p* = 0.590; Figure 2A), ADAS13 (*p* = 0.844; Figure 2F), or CDR‐SB (*p* = 0.366; Figure 2K). Congruent results were obtained in A4/LEARN, with absent associations between Centiloid changes and changes in MMSE (*p* = 0.425; Figure 3A) or PACC (*p* = 0.462; Figure 3F). Regional analyses further confirmed this finding of an absent association between amyloid‐PET changes and parallel cognitive changes (Figure 4A and Figure S1 for a breakdown by clinical group in ADNI).

In contrast, longitudinal changes in temporal meta tau‐PET, plasma p‐tau_217_, and AD signature cortical thickness were consistently associated with cognitive change rates across all cognitive measures in ADNI, including the MMSE (tau‐PET: *β* = −0.100, *p* = 0.001; Figure 2B; plasma p‐tau_217_: *β* = −0.215, *p* = 0.005; Figure 2C; cortical thickness: *β* = 0.606, *p* < 0.001, Figure 2D), ADAS13 (tau‐PET: *β* = 0.369, *p* < 0.001, Figure 2G; plasma p‐tau_217_: *β* = 0.131, *p* = 0.004; Figure 2H; cortical thickness: *β* = ‐0.445, *p *< 0.001, Figure 2I), and CDR‐SB (tau‐PET: *β* = 0.292, *p* < 0.001, Figure 2L; plasma p‐tau_217_: *β* = 0.285, *p* < 0.001; Figure 2M; cortical thickness: *β* = −0.463, *p* < 0.001; Figure 2N). All results remained consistent when applying a Bonferroni‐corrected alpha threshold of 0.0125 to correct for four biomarker assessments per cognitive test. Regional mappings showed the strongest associations between tau‐PET increases and faster cognitive decline in temporo‐parietal brain regions (Figure 4B). Similarly, regional mapping of cortical thickness changes showed the strong associations between cortical thinning and faster cognitive decline, with the strongest effects in temporo‐parietal brain regions (Figure 4C). Exploratory subanalyses stratified by cognitive status (Table S1, Figure S1) yielded overall consistent results in CN (*n* = 94) but limited associations between tau pathophysiology biomarkers and cognitive changes in MCI/dementia (*n* = 47), potentially due to limited sample size in the cognitively impaired group. Congruently, in the A4/LEARN cohort, changes in temporal meta tau‐PET, plasma p‐tau_217_, and AD signature cortical thickness were associated with changes in both the MMSE (tau‐PET: *β* = −0.506, *p* < 0.001; Figure 3B; plasma p‐tau_217_: β = −0.238, *p* = 0.002; Figure 3C; cortical thickness: *β* = 0.553, *p *< 0.001; Figure 3D) and PACC (tau‐PET: *β* = −0.553, *p* < 0.001; Figure 3G; plasma p‐tau_217_: *β *= −0.234, *p* = 0.002; Figure 3H; cortical thickness: *β* = 0.502, *p *< 0.001; Figure 3I). Again, all results remained consistent after Bonferroni correction. In A4/LEARN, regional mapping showed strong associations between faster tau‐PET increase, faster cortical thinning, and more rapid cognitive decline in temporo‐parietal and frontal regions (Figure 4B,C). All results remained consistent when adjusting for ApoE ε4 status (Table S2). Together, these results support the view that longitudinal changes in tau‐PET, plasma p‐tau_217_, and cortical thickness track concurrent cognitive decline, whereas Centiloid changes do not. These findings have important clinical implications, suggesting that amyloid increase does not track cognitive decline, while downstream biomarker changes of tau pathophysiology and neurodegeneration do. Detailed regression statistics are summarized in Table 2.

**TABLE 2:** Regression‐derived associations between rates of changes in biomarker levels and cognition.

ADNI (*n* = 141)					
Dependent variable	Predictor	*β* [bootstrapped 95% CI]	*T*	*p*	Partial *R* ^2^
MMSE ROC	Centiloid ROC^a^	−0.043 [−0.241; 0.153]	−0.540	0.590	0.002
	Temporal meta tau‐PET ROC^a^	−0.100 [−0.192; −0.027]	−3.25	0.001^*^	0.074
	Plasma p‐tau_217_ ROC^a^	−0.215 [−0.397; −0.054]	−2.869	0.005^*^	0.058
	Cortical thickness meta ROC^b^	0.606 [0.408; 0.749]	8.303	<0.001^*^	0.343
ADAS13 ROC	Centiloid ROC^a^	−0.016 [−0.201; 0.182]	−0.197	0.844	<0.001
	Temporal meta tau‐PET ROC^a^	0.369 [0.193; 0.529]	5.918	<0.001^*^	0.208
	Plasma p‐tau_217_ ROC^a^	0.131 [0.049; 0.219]	2.870	0.004^*^	0.058
	Cortical thickness meta ROC^b^	−0.445 [−0.573; −0.238]	−6.366	<0.001^*^	0.234
CDR‐SB ROC	Centiloid ROC^a^	0.069 [−0.084; 0.230]	0.907	0.366	0.006
	Temporal meta tau‐PET ROC^a^	0.292 [0.093; 0.518]	3.529	<0.001^*^	0.086
	Plasma p‐tau_217_ ROC^a^	0.285 [0.104; 0.476]	3.414	<0.001^*^	0.081
	Cortical thickness meta ROC^b^	−0.463 [−0.615; −0.189]	−6.583	<0.001^*^	0.246

A4/LEARN (*n* = 151)					
Dependent variable	Predictor	β [bootstrapped 95% CI]	*T*	*p*	Partial *R* ^2^
MMSE ROC	Centiloid ROC^c^	0.065 [−0.108; −0.219]	0.801	0.425	0.004
	Temporal meta tau‐PET ROC^c^	−0.506 [−0.619; −0.354]	−7.854	<0.001^*^	0.300
	Plasma p‐tau_217_ ROC^c^	−0.238 [−0.416; −0.020]	−3.234	0.002^*^	0.068
	Cortical thickness meta ROC^d^	0.553 [0.387; 0.682]	8.592	<0.001^*^	0.334
PACC ROC	Centiloid ROC^c^	0.057 [−0.116; 0.232]	0.738	0.462	0.004
	Temporal meta tau‐PET ROC^c^	−0.553 [−0.664; −0.432]	−9.254	<0.001^*^	0.373
	Plasma p‐tau_217_ ROC^c^	−0.234 [−0.427; −0.039]	−3.243	0.002^*^	0.068
	Cortical thickness meta ROC^d^	0.502 [0.340; 0.638]	7.785	<0.001^*^	0.296

^a^ Age, sex, education, clinical status, maximum follow‐up duration, and baseline cognition included as covariates.

^b^ Age, sex, education, clinical status, maximum follow‐up duration, total intracranial volume, and baseline cognition included as covariates.

^c^ Age, sex, education, maximum follow‐up duration, and baseline cognition included as covariates.

^d^ Age, sex, education, maximum follow‐up duration, total intracranial volume, and baseline cognition included as covariates.

* Significant after Bonferroni correction (adjusted alpha threshold for four tests = 0.0125).

Abbreviations: Aβ, amyloid beta; AD, Alzheimer's disease; ADAS13, Alzheimer's Disease Assessment Scale‐Cognitive Subscale 13‐item version; ADNI, Alzheimer's Disease Neuroimaging Initiative; CDR‐SB, Clinical Dementia Rating ‐ sum of boxes; CI, confidence interval; MCI, mild cognitive impairment; MMSE, Mini‐Mental State Examination; PACC, Preclinical Alzheimer Cognitive Composite; PET, positron emission tomography; p‐tau_217_, phosphorylated tau at threonine 217; ROC, rate of change.

### 3.3 A systematic effect–size comparison o fATN biomarker changes to track cognitive decline

To further compare the ability of biomarker changes to track cognitive changes, we performed non‐parametric comparisons across biomarker change‐based prediction of cognitive changes by computing 95% CIs of standardized beta value distributions. These beta value distributions were derived from 1000 bootstrapped iterations of linear regression models examining the relationship between biomarker changes and cognitive decline, and the 95% CIs were calculated using the percentile method. Consistent with our previous findings, changes in temporal meta tau‐PET, plasma p‐tau_217_, and AD signature cortical thickness remained strong predictors of cognitive decline in ADNI, as indicated by CIs that did not cross zero for changes in the MMSE (tau‐PET: 95% CI = [−0.192; −0.027], plasma p‐tau_217_: 95% CI = [−0.397; −0.054]; cortical thickness: 95% CI = [0.408; 0.749]; Figure 2E), ADAS13 (tau‐PET: 95% CI = [0.193; 0.529]; plasma p‐tau_217_: 95% CI = [0.049; 0.219]; cortical thickness: 95% CI = [−0.573; −0.238]; Figure 2J), and CDR‐SB (tau‐PET: 95% CI = [0.093; 0.518]; plasma p‐tau_217_: 95% CI = [0.104; 0.476]; cortical thickness: 95% CI = [−0.615; −0.189]; Figure 2O). In contrast, the CIs for Centiloid crossed zero, providing non‐parametric evidence for no statistically significant association with cognitive decline (MMSE: 95% CI = [−0.241; 0.153]; Figure 2E; ADAS13: 95% CI = [−0.201; 0.182]; Figure 2J; CDR‐SB: 95% CI = [−0.084; 0.230]; Figure 2O). Similar patterns were observed in A4/LEARN, where changes in temporal meta tau‐PET, plasma p‐tau_217_, and AD signature cortical thickness showed non‐parametric evidence for tracking cognitive changes in the MMSE (tau‐PET: 95% CI = [−0.619; −0.354]; plasma p‐tau_217_: 95% CI = [−0.416; −0.020]; cortical thickness: 95% CI = [0.387; 0.682]; Figure 3E) and PACC (tau‐PET: 95% CI = [−0.664; −0.432]; plasma p‐tau_217_: 95% CI = [−0.427; −0.039]; cortical thickness: 95% CI = [0.340; 0.638]; Figure 3J). As in ADNI, Centiloid was not considered significant, as its CIs crossed zero (MMSE: 95% CI = [−0.108; −0.219]; Figure 3E; PACC: 95% CI = [−0.116; 0.232]; Figure 3J).

Lastly, we systematically compared the effect sizes of each biomarker to track cognitive changes by comparing effect size differences (Cohen's *d*) of bootstrapped absolute beta values for associations between tau‐PET, plasma p‐tau_217_, and cortical thickness changes and cognitive changes. In ADNI, plasma p‐tau_217_ showed superior performance to tau‐PET for MMSE (*d* = 1.74) and ADAS13 (*d*  = 3.50), but not for CDR‐SB (*d* = −0.03), while cortical thickness showed even better performance than plasma p‐tau_217_ for MMSE (*d* = 4.39), ADAS13 (*d *= −4.38) and CDR‐SB (*d* = −1.51). In A4/LEARN, tau‐PET was superior to plasma p‐tau_217_ for MMSE (*d*  = −3.03) and PACC (*d* = −3.79), and slightly superior to MRI‐assessed cortical thickness (MMSE: *d* = 0.68, PACC: *d*  = −0.78). A detailed summary of effect–size comparisons for all biomarker pairs is shown in Table 3. Together, these findings suggest that MRI, tau‐PET, and plasma p‐tau_217_ track cognitive changes, with MRI often outperforming markers of tau pathophysiology, nevertheless all outperforming amyloid‐PET.

**TABLE 3:** Effect sizes using absolute standardized beta values of bootstrapped linear models for association between biomarker modalities and cognitive changes.

*ADNI* (*n *= 141)	MMSE ROC	ADAS13 ROC	CDR‐SB ROC
Centiloid ROC versus tau‐PET ROC	0.73 (medium)	−4.12 (large)	−2.19 (large)
Centiloid ROC versus plasma p‐tau_217_ ROC	1.86 (large)	−1.89 (large)	−2.47 (large)
Centiloid ROC versus cortical thickness ROC	5.88 (large)	−4.78 (large)	−3.90 (large)
Tau‐PET ROC versus plasma p‐tau_217_ ROC	1.74 (large)	3.50 (large)	−0.03 (negligible)
Tau‐PET ROC versus cortical thickness ROC	7.36 (large)	−0.77 (medium)	−1.42 (large)
Plasma p‐tau_217_ ROC versus cortical thickness ROC	4.39 (large)	−4.38 (large)	−1.51 (large)

*A4/LEARN* (*n *= 151)	MMSE ROC	PACC ROC	
Centiloid ROC versus tau‐PET ROC	7.39 (large)	8.22 (large)	
Centiloid ROC versus plasma p‐tau_217_ ROC	3.17 (large)	3.13 (large)	
Centiloid ROC versus cortical thickness ROC	7.73 (large)	6.82 (large)	
Tau‐PET ROC versus plasma p‐tau_217_ ROC	−3.03 (large)	−3.79 (large)	
Tau‐PET ROC versus cortical thickness ROC	0.68 (medium)	−0.78 (medium)	
Plasma p‐tau_217_ ROC versus cortical thickness ROC	3.48 (large)	2.91 (large)	

Abbreviation: ADAS13, Alzheimer's Disease Assessment Scale‐Cognitive Subscale 13‐item version; ADNI, Alzheimer's Disease Neuroimaging Initiative; CDR‐SB, Clinical Dementia Rating ‐ sum of boxes; MMSE, Mini‐Mental State Examination; PACC, preclinial Alzheimer cognitive composite; PET, positron emission tomography; p‐tau_217_, phosphorylated tau at threonine 217; ROC, rate of change.

## 4 DISCUSSION

Our main aim was to assess the utility of longitudinal changes in A/T/N biomarkers to track cognitive decline in AD to identify which biomarkers could serve as surrogates for clinical treatment efficacy in disease‐modifying treatment settings. Across ADNI and A4/LEARN, we showed that changes in tau‐PET, plasma p‐tau_217_, and cortical thickness accurately tracked cognitive changes across multiple cognitive endpoints (i.e., MMSE, ADAS13, CDR‐SB, PACC), while changes in Centiloid did not track cognitive decline, neither globally nor on the regional level. Considering factors such as costs, availability, invasiveness, and ease of implementation, repeated plasma p‐tau_217_ sampling may therefore be the most promising and AD‐specific surrogate biomarker for monitoring cognitive changes in clinical routine, while tau‐PET and MRI are potentially more suitable in clinical trial and study settings.

Aβ‐plaque removal via donanemab and lecanemab has shown beneficial effects on attenuating cognitive decline, especially in early‐stage AD patients with low tau‐PET levels.^50^, ^51^, ^52^ However, despite the central role of Aβ in initiating AD pathophysiology, its utility in monitoring clinical treatment efficacy remains limited, since removal of Aβ does not necessarily translate into a cognitive benefit.^53^, ^54^ While previous studies showed that Aβ levels were less predictive of cognitive decline than tau biomarkers,^11^, ^12^, ^28^, ^55^ our findings go further by demonstrating that longitudinal increases in fibrillar Aβ do not track concurrent cognitive deterioration in AD. Since amyloid‐PET lowering is considered by the FDA a “reasonably likely surrogate endpoint” for clinical treatment efficacy, our findings challenge this view. Here, future studies should investigate whether Aβ removal is a reliable marker for tracking cognitive changes in anti‐Aβ‐treated patients, or whether the removal of Aβ translates into attenuated cognitive decline by indirectly slowing downstream processes such as tau pathophysiology and neurodegeneration, which track cognitive decline much more closely. This is critical because no T or N biomarkers qualify for the FDA's Accelerated Approval Program.^56^ Thus, there is a key need for validated and potentially more suitable AD biomarkers that signal clinical treatment efficacy in AD trials.^57^

In contrast to amyloid‐PET, tau‐PET changes demonstrated strong associations with cognitive decline. This is in line with the high prognostic utility of tau‐PET for future cognitive changes.^11^, ^27^, ^28^, ^58^ The regional patterns of longitudinal tau‐PET increases that track cognitive decline aligns with tau‐PET staging models of AD.^47^, ^59^ This supports the growing consensus that especially temporal lobe tau‐PET is a strong candidate for tracking both pathophysiological and clinical AD progression.^55^, ^60^, ^61^ However, beyond its cross‐sectional diagnostic use, the use of longitudinal tau‐PET in clinical routine settings is potentially limited by high costs, restricted availability, and radiation exposure for repeated assessments. These factors underscore the need for more accessible, cost‐effective AD‐specific alternatives that can accurately track concurrent clinical changes. In this context, our results confirm the role of plasma p‐tau_217_ as a promising and highly AD‐specific alternative to tau‐PET in clinical settings. Plasma p‐tau_217_ is highly sensitive for detecting AD pathophysiology, showing a strong association with fibrillar Aβ and tau markers as well as CSF‐derived markers of tau pathophysiology.^62^, ^63^, ^64^ As a key advantage, plasma biomarkers are less invasive, easier to obtain in clinical routine, and more suitable for repeated sampling than CSF. Therefore, our findings support previous arguments on plasma p‐tau_217_ as a potential surrogate treatment monitoring tool.^57^ Specifically, we confirmed that plasma p‐tau_217_ changes exhibited strong correlations with cognitive decline, comparable to tau‐PET in ADNI, while p‐tau_217_’s precision lagged that of tau‐PET and cortical thickness in A4 A4/LEARN. This subtle drop in performance might relate to the use of different assays in ADNI versus A4/LEARN or differences in cohort composition, with the A4/LEARN cohort only including cognitively normal Aβ−/Aβ+ participants.^65^ However, a previous study including BioFINDER study and the Wisconsin Registry for Alzheimer Prevention of cognitively normal Aβ+ patients showed high performance of plasma p‐tau_217_ compared to plasma p‐tau_181_, p‐tau_231_, glial fibrillary acidic protein (GFAP), and neurofilament light (NfL) to predict longitudinal changes over 6 years for the MMSE and PACC.^66^ Future studies should therefore assess the accuracy of plasma p‐tau_217_ changes to track cognitive decline across different disease stages to test whether it performs equally well across preclinical and clinical AD stages, which we only covered with exploratory subanalyses stratified by clinical status in ADNI. However, given its accessibility, cost‐effectiveness, and suitability for repeated sampling, plasma p‐tau_217_ could serve as a practical biomarker for both clinical trials and real‐world monitoring of AD progression in observational and potentially treatment settings. These findings suggest that while tau‐PET remains the gold standard for capturing fibrillar tau pathology for diagnostic and prognostic purposes,^11^, ^27^, ^28^, ^58^ plasma p‐tau_217_ presents a viable, less invasive alternative for highly specific monitoring of pathophysiological and clinical AD progression.

Lastly, we examined MRI as a gold‐standard neurodegeneration marker to track clinical changes. Here, cortical thinning was strongly associated with cognitive decline, particularly in AD‐vulnerable temporo‐parietal regions,^67^ often outperforming p‐tau_217_ and tau‐PET. Nevertheless, MRI‐based atrophy metrics in anti‐Aβ trials are confounded by pseudo‐atrophy, potentially reflecting Aβ‐clearance‐related volume reductions, changes in inflammation, and fluid shifts due to treatment that may mimic gray matter atrophy.^68^, ^69^ Drug‐induced increases in ventricular volume and associated brain mass loss were observed across all recent anti‐Aβ treatments.^70^ However, whether this volume loss reflects neurodegeneration^71^ or a side effect of Aβ removal^68^ remains debated.^69^ Given these limitations, MRI‐assessed cortical thickness changes may be non‐ideal for treatment monitoring in anti‐Aβ‐antibody trials, despite their ability to track cognitive changes in untreated AD patients. Still, monitoring brain atrophy has strong potential to monitor natural AD progression or may be suitable for other disease‐modifying drugs (e.g., small molecules, anti‐tau antibodies) that may not induce pseudo‐atrophy.

Conceptually, the identification of suitable biomarkers for tracking cognitive changes in AD requires understanding the cascading pathophysiological events that characterize AD. Aβ deposition initiates AD, occurs early, and slows gradually after reaching the positivity threshold,^72^ thereby potentially limiting its utility as a dynamic marker of clinical progression, which is clearly supported by our findings. While amyloid‐PET remains essential for confirming Aβ positivity in anti‐Aβ trials and for establishing target engagement, it may not be suitable for tracking clinical treatment response, calling into question its role as a reasonably likely surrogate endpoint. In turn, Aβ accumulation triggers p‐tau secretion^73^ and tau aggregation, which are strongly linked to neurodegeneration^67^ and cognitive deterioration.^2^ Here, p‐tau abnormality precedes fibrillar tau aggregation detectable on tau‐PET and predicts future tau aggregation.^74^, ^75^ Thus, p‐tau reflects an intermediate marker of Aβ‐induced tau pathophysiology that promotes downstream tau fibrillization detectable on tau‐PET and, ultimately, neurodegeneration that manifests as cortical thinning on MRI. Therefore, this temporal sequence of biomarker changes aligns with our findings, showing biomarker changes downstream of Aβ are linked to cognitive changes, while Aβ dynamics themselves are not. This distinction is important, as extensive tau pathology limits the clinical efficacy of anti‐Aβ therapies.^52^

A key strength of our study is the use of multiple A/T/N biomarkers to benchmark which biomarker best tracks cognitive decline in AD across two independent cohorts. However, certain caveats should be acknowledged. Treatment effects and natural disease progression are distinct processes, and further research is needed to investigate whether the observed associations between biomarker on cognitive changes also apply for patients undergoing disease‐modifying treatments (e.g., anti‐Aβ). Additionally, while we highlight plasma p‐tau_217_ as a cost‐effective alternative to tau‐PET, its application as a surrogate of cognitive changes remains relatively new. Although our cohorts had follow‐up periods of approximately 5 years, assessing the longitudinal stability of plasma p‐tau_217_ predictions, especially across different clinical disease stages, is crucial for establishing its robustness as a disease‐ and potentially treatment‐monitoring tool. Nevertheless, plasma p‐tau_217_ has the potential to serve as a bridging tool between Aβ deposition and tau accumulation. It can track cognitive changes and potentially be detected before tau‐associated neuronal damage occurs. However, it will also be important to establish a systematic comparison across plasma p‐tau_217_ and other CSF‐based p‐tau markers (which were not consistently available across ADNI and A4/LEARN) as surrogates for clinical progression in AD, to fully understand the performance of p‐tau_217_ as a fluid marker of tau pathophysiology. Further, different biomarkers had different follow‐up durations within the studied cohorts, which may influence the overall estimation of annual change rates. While adjusting for maximum follow‐up duration in our statistical models, our findings should be replicated across fixed observation intervals in future studies. Finally, ADNI and A4/LEARN predominantly include individuals of White ethnicity. To generalize our findings to a broader AD population, replication in more diverse ethnic groups is essential.

Together, our findings emphasize the need for carefully chosen AD biomarkers to track disease progression and potential treatment responses beyond Aβ clearance. While amyloid‐PET aids diagnosis and participant selection for anti‐Aβ treatment, its weak link to cognitive changes calls for alternatives. Tau‐PET, plasma p‐tau_217_, and cortical thickness consistently tracked cognitive changes. While tau‐PET and cortical thickness are contenders, the high cost of tau‐PET and the limitations of cortical thickness (i.e., pseudo‐atrophy) highlight plasma p‐tau_217_ as a practical and cost‐effective alternative. As such, plasma p‐tau_217_ holds strong potential for longitudinal monitoring and could help optimize clinical trial design and therapeutic strategies.

## CONFLICT OF INTEREST STATEMENT

Declarations of interest: none. Author disclosures are available in the supporting information.

## CONSENT STATEMENT

All study procedures were in accordance with the Declaration of Helsinki. Ethical approval was obtained by ADNI and A4/LEARN investigators, and all study participants provided written informed consent.

## Supporting information



Supporting Information

Supporting Information

## References

[1] Jack CR Jr , Bennett DA , Blennow K , et al. NIA‐AA Research Framework: toward a biological definition of Alzheimer's disease. Alzheimers Dement. 2018;14(4):535‐562. doi:10.1016/j.jalz.2018.02.018 29653606 PMC5958625

[2] Jack CR Jr , Knopman DS , Jagust WJ , et al. Hypothetical model of dynamic biomarkers of the Alzheimer's pathological cascade. Lancet Neurol. 2010;9(1):119‐128. doi:10.1016/S1474-4422(09)70299-6 20083042 PMC2819840

[3] Jia J , Ning Y , Chen M , et al. Biomarker changes during 20 years preceding Alzheimer's disease. N Engl J Med. 2024;390(8):712‐722. doi:10.1056/NEJMoa2310168 38381674

[4] Mullard A . FDA approves third anti‐amyloid antibody for Alzheimer disease. Nat Rev Drug Discov. 2024;23(8):571. doi:10.1038/d41573-024-00116-1 38969747

[5] Ramanan VK , Armstrong MJ , Choudhury P , et al. Antiamyloid monoclonal antibody therapy for Alzheimer disease: emerging issues in neurology. Neurology. 2023;101(19):842‐852. doi:10.1212/WNL.0000000000207757 37495380 PMC10663011

[6] Huang LK , Kuan YC , Lin HW , Hu CJ . Clinical trials of new drugs for Alzheimer disease: a 2020‐2023 update. J Biomed Sci. 2023;30(1):83. doi:10.1186/s12929-023-00976-6 37784171 PMC10544555

[7] Jack CR, Jr , Andrews JS , Beach TG , et al. Revised criteria for diagnosis and staging of Alzheimer's disease: Alzheimer's association workgroup. Alzheimers Dement. 2024;20(8):5143‐5169. doi:10.1002/alz.13859 38934362 PMC11350039

[8] Pemberton HG , Collij LE , Heeman F , et al. Quantification of amyloid PET for future clinical use: a state‐of‐the‐art review. Eur J Nucl Med Mol Imaging. 2022;49(10):3508‐3528. doi:10.1007/s00259-022-05784-y 35389071 PMC9308604

[9] Wang G , Li Y , Xiong C , et al. Examining amyloid reduction as a surrogate endpoint through latent class analysis using clinical trial data for dominantly inherited Alzheimer's disease. Alzheimers Dement. 2024;20(4):2698‐2706. doi:10.1002/alz.13735 38400532 PMC11032558

[10] Raket LL , Cummings J , Moscoso A , Villain N , Scholl M . Scenarios for the long‐term efficacy of amyloid‐targeting therapies in the context of the natural history of Alzheimer's disease. Alzheimers Dement. 2024;20(9):6374‐6383. doi:10.1002/alz.14134 39073291 PMC11497713

[11] Biel D , Brendel M , Rubinski A , et al. Tau‐PET and in vivo Braak‐Staging as prognostic markers of future cognitive decline in cognitively normal to demented individuals. Alzheimers Res Ther. 2021;13(1):137. doi:10.1186/s13195-021-00880-x 34384484 PMC8361801

[12] Ioannou K , Bucci M , Tzortzakakis A , et al. Tau PET positivity predicts clinically relevant cognitive decline driven by Alzheimer's disease compared to comorbid cases; proof of concept in the ADNI study. Mol Psychiatry. 2025;30(2):587‐599. doi:10.1038/s41380-024-02672-9 39179903 PMC11746147

[13] Braak H , Braak E . Neuropathological staging of Alzheimer‐related changes. Acta Neuropathol. 1991;82(4):239‐259. doi:10.1007/bf00308809 1759558

[14] Chen SD , Lu JY , Li HQ , et al. Staging tau pathology with tau PET in Alzheimer's disease: a longitudinal study. Transl Psychiatry. 2021;11(1):483. doi:10.1038/s41398-021-01602-5 34537810 PMC8449785

[15] Therriault J , Pascoal TA , Lussier FZ , et al. Biomarker modeling of Alzheimer's disease using PET‐based Braak staging. Nat Aging. 2022;2(6):526‐535. doi:10.1038/s43587-022-00204-0 37118445 PMC10154209

[16] Lai R , Li B , Bishnoi R . P‐tau217 as a reliable blood‐based marker of Alzheimer's disease. Biomedicines. 2024;12(8):1836. doi:10.3390/biomedicines12081836 39200300 PMC11351463

[17] Thijssen EH , La Joie R , Strom A , et al. Plasma phosphorylated tau 217 and phosphorylated tau 181 as biomarkers in Alzheimer's disease and frontotemporal lobar degeneration: a retrospective diagnostic performance study. Lancet Neurol. 2021;20(9):739‐752. doi:10.1016/S1474-4422(21)00214-3 34418401 PMC8711249

[18] Karikari TK , Benedet AL , Ashton NJ , et al. Diagnostic performance and prediction of clinical progression of plasma phospho‐tau181 in the Alzheimer's Disease Neuroimaging Initiative. Mol Psychiatry. 2021;26(2):429‐442. doi:10.1038/s41380-020-00923-z 33106600

[19] Mattsson‐Carlgren N , Janelidze S , Bateman RJ , et al. Soluble P‐tau217 reflects amyloid and tau pathology and mediates the association of amyloid with tau. EMBO Mol Med. 2021;13(6):e14022. doi:10.15252/emmm.202114022 33949133 PMC8185545

[20] Therriault J , Ashton NJ , Pola I , et al. Comparison of two plasma p‐tau217 assays to detect and monitor Alzheimer's pathology. EBioMedicine. 2024;102:105046. doi:10.1016/j.ebiom.2024.105046 38471397 PMC10943661

[21] Barthelemy NR , Horie K , Sato C , Bateman RJ . Blood plasma phosphorylated‐tau isoforms track CNS change in Alzheimer's disease. J Exp Med. 2020;217(11):e20200861. doi:10.1084/jem.20200861 32725127 PMC7596823

[22] Querbes O , Aubry F , Pariente J , et al. Early diagnosis of Alzheimer's disease using cortical thickness: impact of cognitive reserve. Brain. 2009;132(Pt 8):2036‐2047. doi:10.1093/brain/awp105 19439419 PMC2714060

[23] Frisoni GB , Fox NC , Jack CR, Jr , Scheltens P , Thompson PM . The clinical use of structural MRI in Alzheimer disease. Nat Rev Neurol. 2010;6(2):67‐77. doi:10.1038/nrneurol.2009.215 20139996 PMC2938772

[24] Ossenkoppele R , Smith R , Ohlsson T , et al. Associations between tau, Abeta, and cortical thickness with cognition in Alzheimer disease. Neurology. 2019;92(6):e601‐e612. doi:10.1212/WNL.0000000000006875 30626656 PMC6382060

[25] Velayudhan L , Proitsi P , Westman E , et al. Entorhinal cortex thickness predicts cognitive decline in Alzheimer's disease. J Alzheimers Dis. 2013;33(3):755‐766. doi:10.3233/JAD-2012-121408 23047370

[26] Kim J , Kim J , Park YH , et al. Distinct spatiotemporal patterns of cortical thinning in Alzheimer's disease‐type cognitive impairment and subcortical vascular cognitive impairment. Commun Biol. 2024;7(1):198. doi:10.1038/s42003-024-05787-5 38368479 PMC10874406

[27] Ossenkoppele R , Pichet Binette A , Groot C , et al. Amyloid and tau PET‐positive cognitively unimpaired individuals are at high risk for future cognitive decline. Nat Med. 2022;28(11):2381‐2387. doi:10.1038/s41591-022-02049-x 36357681 PMC9671808

[28] Ossenkoppele R , Smith R , Mattsson‐Carlgren N , et al. Accuracy of Tau positron emission tomography as a prognostic marker in preclinical and prodromal Alzheimer disease: a head‐to‐head comparison against amyloid positron emission tomography and magnetic resonance imaging. JAMA Neurol. 2021;78(8):961‐971. doi:10.1001/jamaneurol.2021.1858 34180956 PMC8240013

[29] Ossenkoppele R , Salvado G , Janelidze S , et al. Plasma p‐tau217 and tau‐PET predict future cognitive decline among cognitively unimpaired individuals: implications for clinical trials. Nat Aging. 2025;5(5):883‐896. doi:10.1038/s43587-025-00835-z 40155777 PMC12092243

[30] Biel D , Suarez‐Calvet M , Dewenter A , et al. Female sex is linked to a stronger association between sTREM2 and CSF p‐tau in Alzheimer's disease. EMBO Mol Med. 2025;17(2):235‐248. doi:10.1038/s44321-024-00190-3 39794447 PMC11822105

[31] Roemer‐Cassiano SN , Wagner F , Evangelista L , et al. Amyloid‐associated hyperconnectivity drives tau spread across connected brain regions in Alzheimer's disease. Sci Transl Med. 2025;17(782):eadp2564. doi:10.1126/scitranslmed.adp2564 39841807

[32] Landau SM , Mintun MA , Joshi AD , et al. Amyloid deposition, hypometabolism, and longitudinal cognitive decline. Ann Neurol. 2012;72(4):578‐586. doi:10.1002/ana.23650 23109153 PMC3786871

[33] Royse SK , Minhas DS , Lopresti BJ , et al. Validation of amyloid PET positivity thresholds in centiloids: a multisite PET study approach. Alzheimers Res Ther. 2021;13(1):99. doi:10.1186/s13195-021-00836-1 33971965 PMC8111744

[34] Folstein MF , Folstein SE , McHugh PR . “Mini‐mental state”. A practical method for grading the cognitive state of patients for the clinician. J Psychiatr Res. 1975;12(3):189‐198. doi:10.1016/0022-3956(75)90026-6 1202204

[35] Skinner J , Carvalho JO , Potter GG , et al. The Alzheimer's Disease Assessment Scale‐Cognitive‐Plus (ADAS‐Cog‐Plus): an expansion of the ADAS‐Cog to improve responsiveness in MCI. Brain Imaging Behav. 2012;6(4):489‐501. doi:10.1007/s11682-012-9166-3 22614326 PMC3873823

[36] Morris JC . The Clinical Dementia Rating (CDR): current version and scoring rules. Neurology. 1993;43(11):2412‐2414. doi:10.1212/wnl.43.11.2412-a 8232972

[37] Donohue MC , Sperling RA , Salmon DP , et al. The preclinical Alzheimer cognitive composite: measuring amyloid‐related decline. JAMA Neurol. 2014;71(8):961‐970. doi:10.1001/jamaneurol.2014.803 24886908 PMC4439182

[38] Mormino EC , Papp KV , Rentz DM , et al. Early and late change on the preclinical Alzheimer's cognitive composite in clinically normal older individuals with elevated amyloid beta. Alzheimers Dement. 2017;13(9):1004‐1012. doi:10.1016/j.jalz.2017.01.018 28253478 PMC5573651

[39] Shanks HRC , Chen K , Reiman EM , et al. p75 neurotrophin receptor modulation in mild to moderate Alzheimer disease: a randomized, placebo‐controlled phase 2a trial. Nat Med. 2024;30(6):1761‐1770. doi:10.1038/s41591-024-02977-w 38760589 PMC11186782

[40] Rentz DM , Rosenberg PB , Sperling RA , et al. Characterizing clinical progression in cognitively unimpaired older individuals with brain amyloid: results from the A4 study. J Prev Alzheimers Dis. 2024;11(4):814‐822. doi:10.14283/jpad.2024.123 39044489 PMC11266445

[41] Rissman RA , Donohue MC , Langford O , et al. Longitudinal Phospho‐tau217 Predicts amyloid positron emission tomography in asymptomatic Alzheimer's disease. J Prev Alzheimers Dis. 2024;11(4):823‐830. doi:10.14283/jpad.2024.134 39044490 PMC11266279

[42] Jack CR, Jr , Wiste HJ , Weigand SD , et al. Defining imaging biomarker cut points for brain aging and Alzheimer's disease. Alzheimers Dement. 2017;13(3):205‐216. doi:10.1016/j.jalz.2016.08.005 27697430 PMC5344738

[43] Landau SM , Thomas BA , Thurfjell L , et al. Amyloid PET imaging in Alzheimer's disease: a comparison of three radiotracers. Eur J Nucl Med Mol Imaging. 2014;41(7):1398‐1407. doi:10.1007/s00259-014-2753-3 24647577 PMC4055504

[44] Baker SL , Maass A , Jagust WJ . Considerations and code for partial volume correcting [(18)F]‐AV‐1451 tau PET data. Data Brief. 2017;15:648‐657. doi:10.1016/j.dib.2017.10.024 29124088 PMC5671473

[45] Schaefer A , Kong R , Gordon EM , et al. Local‐Global parcellation of the Human cerebral cortex from intrinsic functional connectivity MRI. Cereb Cortex. 2018;28(9):3095‐3114. doi:10.1093/cercor/bhx179 28981612 PMC6095216

[46] Klunk WE , Koeppe RA , Price JC , et al. The Centiloid Project: standardizing quantitative amyloid plaque estimation by PET. Alzheimers Dement. 2015;11(1):1‐15.e1‐4. doi:10.1016/j.jalz.2014.07.003 25443857 PMC4300247

[47] Scholl M , Lockhart SN , Schonhaut DR , et al. PET imaging of tau deposition in the aging human brain. Neuron. 2016;89(5):971‐982. doi:10.1016/j.neuron.2016.01.028 26938442 PMC4779187

[48] Biel D , Luan Y , Brendel M , et al. Combining tau‐PET and fMRI meta‐analyses for patient‐centered prediction of cognitive decline in Alzheimer's disease. Alzheimers Res Ther. 2022;14(1):166. doi:10.1186/s13195-022-01105-5 36345046 PMC9639286

[49] R: A language and environment for statistical computing . R Foundation for Statistical Computing; 2021. https://www.R‐project.org/

[50] van Dyck CH , Swanson CJ , Aisen P , et al. Lecanemab in early Alzheimer's disease. N Engl J Med. 2023;388(1):9‐21. doi:10.1056/NEJMoa2212948 36449413

[51] Mintun MA , Lo AC , Duggan Evans C , et al. Donanemab in early Alzheimer's disease. N Engl J Med. 2021;384(18):1691‐1704. doi:10.1056/NEJMoa2100708 33720637

[52] Sims JR , Zimmer JA , Evans CD , et al. Donanemab in early symptomatic Alzheimer disease: the TRAILBLAZER‐ALZ 2 randomized clinical trial. JAMA. 2023;330(6):512‐527. doi:10.1001/jama.2023.13239 37459141 PMC10352931

[53] Musiek ES , Bennett DA . Aducanumab and the “post‐amyloid” era of Alzheimer research?. Neuron. 2021;109(19):3045‐3047. doi:10.1016/j.neuron.2021.09.007 34582783 PMC9308468

[54] Kuller LH , Lopez OL . ENGAGE and EMERGE: truth and consequences?. Alzheimers Dement. 2021;17(4):692‐695. doi:10.1002/alz.12286 33656288 PMC8248059

[55] Bucci M , Chiotis K , Nordberg A . Alzheimer's Disease neuroimaging i. Alzheimer's disease profiled by fluid and imaging markers: tau PET best predicts cognitive decline. Mol Psychiatry. 2021;26(10):5888‐5898. doi:10.1038/s41380-021-01263-2 34593971 PMC8758489

[56] US Food and Drug Administration . Surrogate Endpoint Resources for Drug and Biologic Development. Accessed 21.03.2025, https://www.fda.gov/drugs/development‐resources/surrogate‐endpoint‐resources‐drug‐and‐biologic‐development

[57] Hansson O , Blennow K , Zetterberg H , Dage J . Blood biomarkers for Alzheimer's disease in clinical practice and trials. Nat Aging. 2023;3(5):506‐519. doi:10.1038/s43587-023-00403-3 37202517 PMC10979350

[58] Moscoso A , Heeman F , Raghavan S , et al. Frequency and clinical outcomes associated with tau positron emission tomography positivity. JAMA. 2025;334(3):229‐242. doi:10.1001/jama.2025.7817 40522652 PMC12848857

[59] Franzmeier N , Dewenter A , Frontzkowski L , et al. Patient‐centered connectivity‐based prediction of tau pathology spread in Alzheimer's disease. Sci Adv. 2020;6(48). doi:10.1126/sciadv.abd1327 PMC7695466 33246962

[60] Groot C , Smith R , Collij LE , et al. Tau Positron emission tomography for predicting dementia in individuals with mild cognitive impairment. JAMA Neurol. 2024;81(8):845‐856. doi:10.1001/jamaneurol.2024.1612 38857029 PMC11165418

[61] Groot C , Villeneuve S , Smith R , Hansson O , Ossenkoppele R . Tau PET imaging in neurodegenerative disorders. J Nucl Med. 2022;63(Suppl 1):20S‐26S. doi:10.2967/jnumed.121.263196 35649647

[62] Janelidze S , Stomrud E , Smith R , et al. Cerebrospinal fluid p‐tau217 performs better than p‐tau181 as a biomarker of Alzheimer's disease. Nat Commun. 2020;11(1):1683. doi:10.1038/s41467-020-15436-0 32246036 PMC7125218

[63] Leuzy A , Janelidze S , Mattsson‐Carlgren N , et al. Comparing the clinical utility and diagnostic performance of CSF P‐Tau181, P‐Tau217, and P‐Tau231 Assays. Neurology. 2021;97(17):e1681‐e1694. doi:10.1212/WNL.0000000000012727 34493616 PMC8605616

[64] Janelidze S , Bali D , Ashton NJ , et al. Head‐to‐head comparison of 10 plasma phospho‐tau assays in prodromal Alzheimer's disease. Brain. 2023;146(4):1592‐1601. doi:10.1093/brain/awac333 36087307 PMC10115176

[65] Palmqvist S , Stomrud E , Cullen N , et al. An accurate fully automated panel of plasma biomarkers for Alzheimer's disease. Alzheimers Dement. 2023;19(4):1204‐1215. doi:10.1002/alz.12751 35950735 PMC9918613

[66] Mattsson‐Carlgren N , Salvado G , Ashton NJ , et al. Prediction of longitudinal cognitive decline in preclinical Alzheimer disease using plasma biomarkers. JAMA Neurol. 2023;80(4):360‐369. doi:10.1001/jamaneurol.2022.5272 36745413 PMC10087054

[67] La Joie R , Visani AV , Baker SL , et al. Prospective longitudinal atrophy in Alzheimer's disease correlates with the intensity and topography of baseline tau‐PET. Sci Transl Med. 2020;12(524):eaau5732. doi:10.1126/scitranslmed.aau5732 31894103 PMC7035952

[68] Belder CRS , Boche D , Nicoll JAR , et al. Brain volume change following anti‐amyloid beta immunotherapy for Alzheimer's disease: amyloid‐removal‐related pseudo‐atrophy. Lancet Neurol. 2024;23(10):1025‐1034. doi:10.1016/S1474-4422(24)00335-1 39304242

[69] Belder CRS , Boche D , Nicoll JAR , et al. Brain volume change following anti‐amyloid β immunotherapy for Alzheimer's disease: amyloid‐removal‐related pseudo‐atrophy. The Lancet Neurology. 2024;23(10):1025‐1034. doi:10.1016/S1474-4422(24)00335-1 39304242

[70] Alves F , Kalinowski P , Ayton S . Accelerated brain volume loss caused by anti‐beta‐amyloid drugs: a systematic review and meta‐analysis. Neurology. 2023;100(20):e2114‐e2124. doi:10.1212/WNL.0000000000207156 36973044 PMC10186239

[71] Couzin‐Frankel J . Promising Alzheimer's therapies shrink brains. Science. 2023;380(6640):19. doi:10.1126/science.adi1220 37023200

[72] Jagust WJ , Landau SM . Alzheimer's disease neuroimaging i. temporal dynamics of beta‐amyloid accumulation in aging and Alzheimer disease. Neurology. 2021;96(9):e1347‐e1357. doi:10.1212/WNL.0000000000011524 33408147 PMC8055327

[73] Sato C , Barthelemy NR , Mawuenyega KG , et al. Tau kinetics in neurons and the human central nervous system. Neuron. 2018;97(6):1284‐1298.e7. doi:10.1016/j.neuron.2018.02.015 29566794 PMC6137722

[74] Pichet Binette A , Franzmeier N , Spotorno N , et al. Amyloid‐associated increases in soluble tau relate to tau aggregation rates and cognitive decline in early Alzheimer's disease. Nat Commun. 2022;13(1):6635. doi:10.1038/s41467-022-34129-4 36333294 PMC9636262

[75] Groot C , Smith R , Stomrud E , et al. Phospho‐tau with subthreshold tau‐PET predicts increased tau accumulation rates in amyloid‐positive individuals. Brain. 2023;146(4):1580‐1591. doi:10.1093/brain/awac329 36084009 PMC10115173

