# Supplementary material for: A systematic comparison of ATN biomarkers for monitoring longitudinal cognitive changes in Alzheimer's disease
Source: Alzheimers Dement. 2025 Oct 22;21(10):e70783. doi: 10.1002/alz.70783 (PMC12545699; doi:10.1002/alz.70783)
Supplement: Supplementary file 1 — Supporting Information [file ALZ-21-e70783-s001.docx]

**Supplementary Table 1.** Regression results stratified by cognitive status in ADNI

| **ADNI – cognitively normal (n=94)** | | | | | |
| --- | --- | --- | --- | --- | --- |
| Dependent Variable | Predictor | β | T | p | Patial R^2^ |
| MMSE ROC | Centiloid ROC^1^ | 0.038 | 0.357 | 0.722 | 0.001 |
|  | Temporal meta tau-PET ROC^1^ | -0.154 | -2.322 | 0.023 | 0.058 |
|  | Plasma p-tau_217_ ROC^1^ | -0.393 | -3.897 | <0.001 | 0.152 |
|  | Cortical thickness meta ROC^2^ | 0.385 | 4.099 | <0.001 | 0.168 |
| ADAS13 ROC^1^ | Centiloid ROC^1^ | -0.104 | -0.986 | 0.327 | 0.011 |
|  | Temporal meta tau-PET ROC^1^ | 0.406 | 4.482 | <0.001 | 0.186 |
|  | Plasma p-tau_217_ ROC^1^ | 0.241 | 2.644 | 0.010 | 0.076 |
|  | Cortical thickness meta ROC^2^ | -0.383 | -3.938 | <0.001 | 0.157 |
| CDR-SB ROC^1^ | Centiloid ROC^1^ | 0.040 | 0.387 | 0.700 | 0.002 |
|  | Temporal meta tau-PET ROC^1^ | 0.238 | 2.297 | 0.024 | 0.057 |
|  | Plasma p-tau_217_ ROC^1^ | 0.404 | 4.632 | <0.001 | 0.202 |
|  | Cortical thickness meta ROC^2^ | -0.487 | -4.985 | <0.001 | 0.230 |
| **ADNI – MCI/Dementia (n=47)** | | | | | |
| Dependent Variable | Predictor | β | T | P | Patial R^2^ |
| MMSE ROC | Centiloid ROC^1^ | -0.137 | -0.842 | 0.405 | 0.017 |
|  | Temporal meta tau-PET ROC^1^ | -0.070 | -1.599 | 0.118 | 0.062 |
|  | Plasma p-tau_217_ ROC^1^ | -0.128 | -0.927 | 0.359 | 0.020 |
|  | Cortical thickness meta ROC^2^ | 0.803 | 7.031 | <0.001 | 0.541 |
| ADAS13 ROC^1^ | Centiloid ROC^1^ | 0.078 | 0.477 | 0.636 | 0.005 |
|  | Temporal meta tau-PET ROC^1^ | 0.352 | 3.111 | 0.003 | 0.199 |
|  | Plasma p-tau_217_ ROC^1^ | 0.139 | 1.901 | 0.064 | 0.079 |
|  | Cortical thickness meta ROC^2^ | -0.559 | -4.549 | <0.001 | 0.330 |
| CDR-SB ROC^1^ | Centiloid ROC^1^ | 0.127 | 0.860 | 0.395 | 0.018 |
|  | Temporal meta tau-PET ROC^1^ | 0.301 | 1.962 | 0.057 | 0.090 |
|  | Plasma p-tau_217_ ROC^1^ | 0.333 | 2.284 | 0.028 | 0.110 |
|  | Cortical thickness meta ROC^2^ | -0.569 | -4.854 | <0.001 | 0.359 |

1. Age, sex, education, maximum follow-up duration and baseline cognition included as covariates
2. Age, sex, education, maximum follow-up duration, total intracranial volume and baseline cognition included as covariates

**Supplementary Table 2.** Regression results adjusted for ApoE4 status

| **ADNI (n=141)** | | | | | |
| --- | --- | --- | --- | --- | --- |
| Dependent Variable | Predictor | β | T | p | Patial R^2^ |
| MMSE ROC | Centiloid ROC^1^ | -0.001 | -0.008 | 0.994 | <0.001 |
|  | Temporal meta tau-PET ROC^1^ | -0.093 | -2.940 | 0.004* | 0.061 |
|  | Plasma p-tau_217_ ROC^1^ | -0.183 | -2.331 | 0.021* | 0.040 |
|  | Cortical thickness meta ROC^2^ | 0.594 | 8.171 | <0.001* | 0.338 |
| ADAS13 ROC | Centiloid ROC^1^ | -0.053 | -0.659 | 0.511 | 0.003 |
|  | Temporal meta tau-PET ROC^1^ | 0.355 | 5.551 | <0.001* | 0.189 |
|  | Plasma p-tau_217_ ROC^1^ | 0.113 | 2.3561 | 0.020* | 0.040 |
|  | Cortical thickness meta ROC^2^ | -0.436 | -6.306 | <0.001* | 0.233 |
| CDR-SB ROC | Centiloid ROC^1^ | 0.026 | 0.340 | 0.734 | <0.001 |
|  | Temporal meta tau-PET ROC^1^ | 0.299 | 3.520 | <0.001* | 0.086 |
|  | Plasma p-tau_217_ ROC^1^ | 0.250 | 2.843 | 0.005* | 0.058 |
|  | Cortical thickness meta ROC^2^ | -0.4491 | -6.495 | <0.001* | 0.244 |
| **A4/LEARN (n=151)** | | | | | |
| Dependent Variable | Predictor | β | T | P | Patial R^2^ |
| MMSE ROC | Centiloid ROC^3^ | 0.086 | 1.010 | 0.314 | 0.007 |
|  | Temporal meta tau-PET ROC^3^ | -0.516 | -7.739 | <0.001* | 0.295 |
|  | Plasma p-tau_217_ ROC^3^ | -0.236 | -3.179 | 0.002* | 0.066 |
|  | Cortical thickness meta ROC^4^ | 0.551 | 8.451 | <0.001* | 0.333 |
| PACC ROC | Centiloid ROC^3^ | 0.072 | 0.884 | 0.378 | 0.005 |
|  | Temporal meta tau-PET ROC^3^ | -0.574 | -9.441 | <0.001* | 0.384 |
|  | Plasma p-tau_217_ ROC^3^ | -0.232 | -3.187 | 0.002* | 0.066 |
|  | Cortical thickness meta ROC^4^ | 0.502 | 7.734 | <0.001* | 0.295 |

1. Age, sex, education, ApoE4 status, maximum follow-up duration, clinical status and baseline cognition included as covariates
2. Age, sex, education, ApoE4 status, maximum follow-up duration, clinical status, total intracranial volume and baseline cognition included as covariates
3. Age, sex, education, maximum follow-up duration, ApoE4 status and baseline cognition included as covariates
4. Age, sex, education, total intracranial volume, maximum follow-up duration, ApoE4 status, and baseline cognition included as covariates

*Significant after Bonferroni correction (adjusted alpha threshold for 4 tests = 0.0125)

**Supplementary figure 1:**


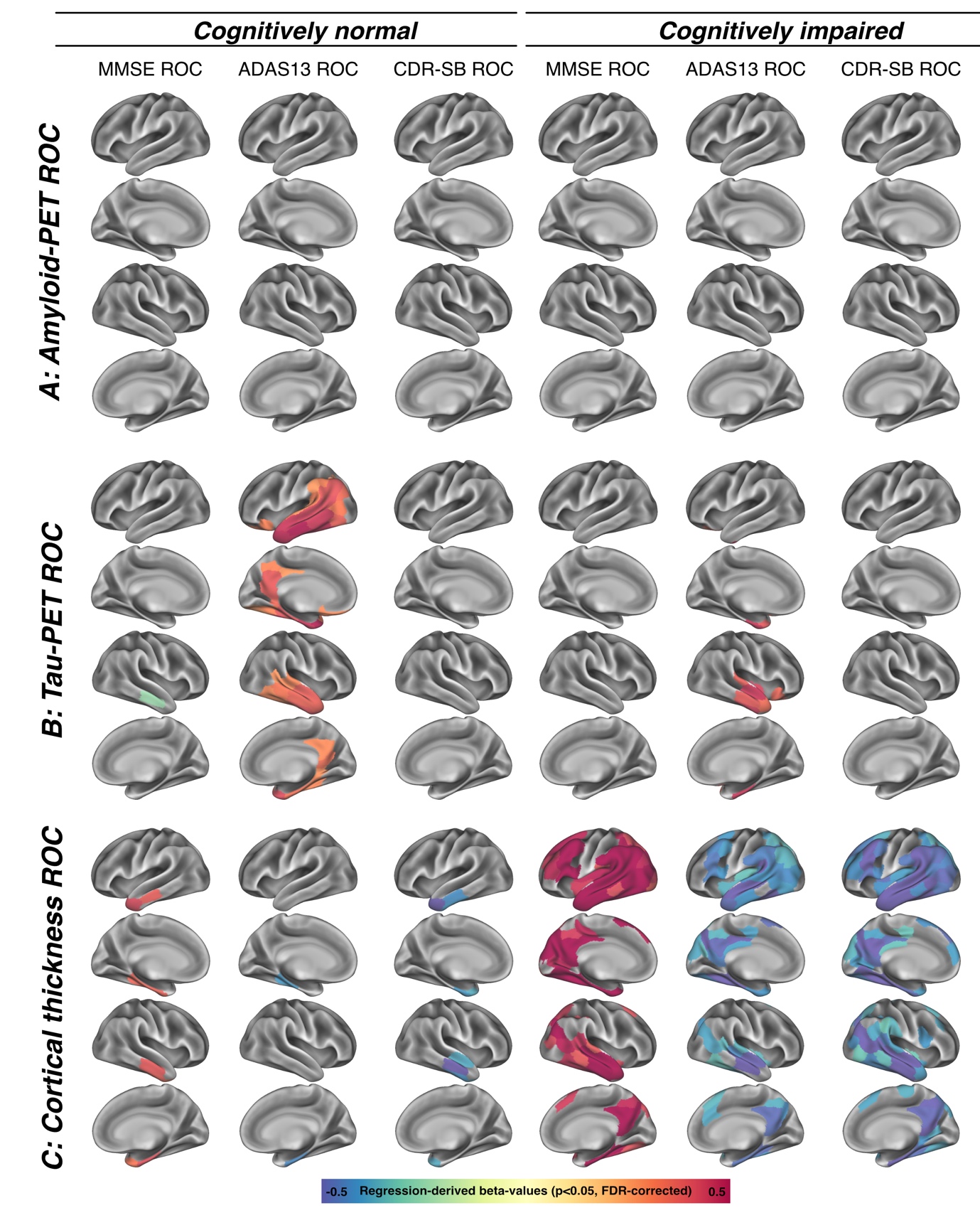


ROI-based regression models, illustrating the associations between annual change rates of amyloid-PET centiloids (A), tau-PET (B) or MRI-assessed cortical thickness (C) with each cognitive test stratified by clinical status in the ADNI cohort. Standardized regression coefficients that were significant (p<0.05) after False-discovery rate (FDR) correction are displayed. All models are controlled for sex, age, education, maximum follow up-duration, baseline cognition and total intracranial volume for cortical thickness analyses.
